# Pterostilbene and resveratrol: Exploring their protective mechanisms against skin photoaging – A scoping review

**DOI:** 10.1016/j.bbrep.2025.102011

**Published:** 2025-04-15

**Authors:** Raveena Vaidheswary Muralitharan, Siti Fathiah Masre, Dayang Fredalina Basri, Ahmad Rohi Ghazali

**Affiliations:** a Center for Toxicology and Health Risk Studies (CORE), Faculty of Health Sciences, Universiti Kebangsaan Malaysia, Kuala Lumpur, Malaysia; b Center for Diagnostic, Therapeutic and Investigative Studies (CODTIS), Faculty of Health Sciences, Universiti Kebangsaan Malaysia, Kuala Lumpur, Malaysia

**Keywords:** Pterostilbene, Resveratrol, Skin, Photoaging

## Abstract

Prolonged ultraviolet (UV) exposure depletes the skin's endogenous antioxidants, leading to photoaging. Exogenous antioxidants are essential to counter this, with stilbenes such as pterostilbene and resveratrol emerging as promising candidates due to their antioxidant, anti-inflammatory and anti-cancer properties. The current scoping review presents an overview of the evidence on the effects of pterostilbene and resveratrol on skin photoaging. A literature search was conducted using PubMed, Scopus, and Web of Science databases in April 2025. Original research articles that investigated the effects of pterostilbene and resveratrol on skin photoaging in cells, animals, or humans were included. 9 eligible articles were included in this review. The findings suggest that resveratrol significantly improves skin photoaging, while preliminary evidence indicates that pterostilbene may offer advantages over resveratrol. However, due to the limited research on pterostilbene, further studies are required to confirm its efficacy. Key considerations in establishing valid in vitro and in vivo models, alongside macroscopic and histologic features of photoaging, were also discussed. In conclusion, while resveratrol shows significant promise in combating skin photoaging, pterostilbene is still in the early exploration phases. Advancing to human trials is crucial to confirm the efficacy of these stilbenes in preventing and treating photoaging.

## Highlights

• Skin photoaging occurs due to prolonged and repeated solar ultraviolet radiation.
• Resveratrol shows promise in combating photoaging in vitro and in vivo.
• Pterostilbene demonstrates potential to outperform resveratrol.
• Standardised models are needed to improve photoaging research reliability.
• Human trials are essential to validate stilbenes' efficacy against photoaging.

## Graphical abstract

**Figure:**
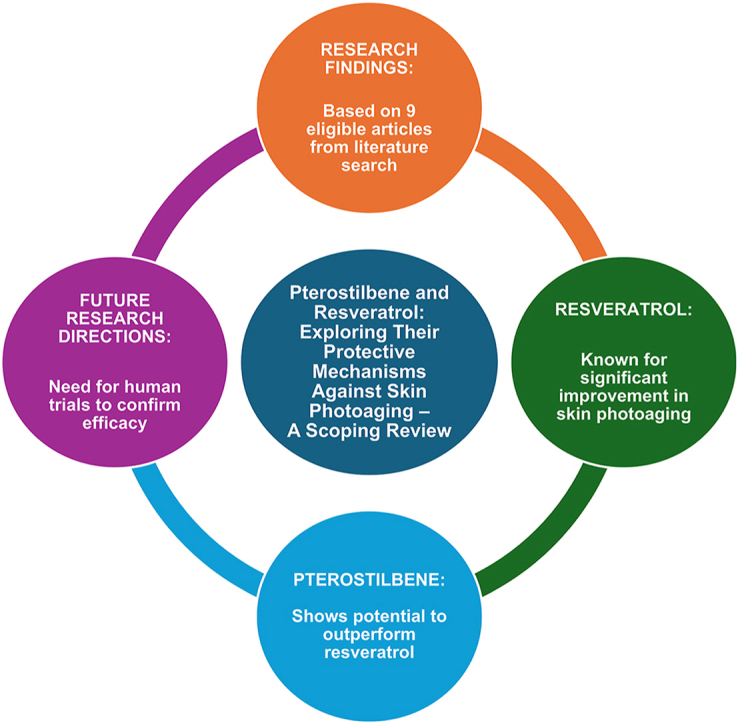

## 1 Introduction

Photoaging is defined as skin damage caused by prolonged and repeated exposure to solar ultraviolet (UV) radiation [[1], [2], [3]]. It is deemed to be a silent disease that affects millions on a global scale [3]. For instance, the occurrence of severe skin photoaging rose from 42 % in 1992 to 88 % in 2007, as observed in a study of predominantly Australian adults that are fair-skinned under the age of 55 living in a sunny Australian climate [4]. There are several factors contributing to these staggering numbers. Recently, a study by Assiri et al. (2024) which investigated knowledge, attitude, reception, and preventive practices of skin photoaging in the Jazan, Saudi Arabia adult population, found that 71.9 % of participants had never heard of photoaging, signalling a critical lack of awareness. The same study also found that 39.16 % of participants admitted to never using sunscreen, further highlighting ignorance toward preventive practices [5]. In addition, lifestyle choices such as smoking, frequent tanning, or occupations involving long hours under the sun [3,6] also increase the susceptibility of individuals towards photoaging.

While the skin is equipped with an antioxidant network comprising of glutathione (GSH), glutathione peroxidase (GPx), superoxide dismutase (SOD) and catalase (CAT) [7], the balance is tipped when oxidative stress from UV radiation overwhelms the skin [8]. The oxidative stress results in skin damage that subsequently leads to photoaging characteristics, such as coarse wrinkles, laxity, fragility, rough skin, telangiectasia, loss of normal complexion, dyspigmentation, and impaired wound healing [[9], [10], [11]]. However, the most concerning consequence of photoaging is its role in advancing precancerous lesions that can ultimately progress to skin cancer due to its DNA mutations and damage [3,12].

In response, the scientific and cosmetics industries are actively building on existing strategies to combat the harmful effects of UV radiation, with a focus on sunscreens, antioxidants and supplements due to the value of a youthful appearance in today's society [3,13,14]. One promising way in decreasing skin oxidative stress and subsequently preventing photoaging involved supplementing the skin with exogenous antioxidants from natural compounds found in plants and fruits [7,15,16]. Ongoing research is diving deeper into understanding the precise mechanisms, pathways and actions of exogenous antioxidants towards photoaging. Ultimately, the aim is to improve the skin's antioxidant defenses against UV-induced photoaging by incorporating antioxidant-rich foods including vegetables and fruits, through diet and supplements, or via topical applications like skincare and sunscreen [3,4].

Among these antioxidants, stilbenes, a class of polyphenols characterised by a core structure of 1,2-diphenylethylene, features 2 benzene rings linked by an ethylene group [17]. With more than 400 naturally occurring stilbenes [18], resveratrol emerged as the most studied stilbene [19]. Resveratrol is a trihydroxy stilbene derivative (3,5,4′-trihydroxystilbene) found in grapes, berries, peanuts and red wine [20]. It is well known for properties, such as anti-aging, anti-inflammatory, anti-melanogenesis, and anti-cancer [[21], [22], [23]]. When it comes to skin photoaging, resveratrol only started gaining traction over the last decade [[24], [25], [26], [27], [28], [29]]. However, resveratrol is sensitive to light and air, which causes the compound to degrade easily and makes it challenging to formulate into effective topical treatments [30]. Besides, resveratrol has a lower bioavailability and shorter half-life than its analogue, pterostilbene [31,32].

Pterostilbene (*trans*-3,5-dimethoxy-4-hydroxystilbene) is found in blueberries [33] and *Pterocarpus marsupium* hardwood [34]. It has anti-aging, anti-melanogenesis and anti-photocarcinogenesis properties [22,35,36]. With two methoxy groups in its chemical structure giving rise to increased lipophilic properties, pterostilbene has the upper hand compared to resveratrol. This advantage increases pterostilbene's bioavailability, extending its half-life and exhibiting greater cellular uptake potential systemically [31].

Despite the extensive research on resveratrol in relation to photoaging, there is still a lack of comprehensive reviews focusing on its molecular targets, mechanisms and pathways. Meanwhile, pterostilbene is chosen for comparison against resveratrol due to its chemical structure advantage and studies highlighting pterostilbene's superiority over resveratrol [[37], [38], [39], [40]]. Additionally, we sought to explore whether pterostilbene holds potential as the next promising stilbene in the field of photoaging by elucidating its molecular targets, mechanisms and pathways. Therefore, the paper aims to analyse the findings from studies that used pterostilbene or resveratrol to understand the mechanism behind mitigating skin photoaging. We hope our review will drive efforts to develop potentially effective anti-photoaging agents and subsequently reduce the risks of developing photoaging and skin cancer.

## 2 Methodology

The scoping review adheres to the Preferred Reporting Items for Systematic Reviews and Meta-Analyses Extension for Scoping Reviews [41] (Supplementary Data). These steps were implemented [1]: identifying the research question [2]; identifying the relevant studies [3]; selecting the studies [4]; charting the data; and [5] reporting the results.

### 2.1 Identifying the research question

The identified research question was: what are the effects of resveratrol and pterostilbene on skin photoaging? The diverse models used to study skin photoaging and its various outcomes were also emphasized in this review.

### 2.2 Identifying the relevant studies

A literature search was conducted using three electronic databases (PubMed, Scopus and Web of Science) in April 2025 using the search string: (resveratrol OR pterostilbene) AND (skin OR dermis OR epidermis OR keratinocytes OR fibroblasts OR melanocytes OR collagen OR elastin OR melanin) AND (photoag∗) AND (ultraviolet OR UV OR UVA OR UVB OR damage). All primary studies which investigated the effect of resveratrol and pterostilbene on skin photoaging were considered. The following types of studies were excluded: books, review articles, non-English publications, nutraceutical combinations and other forms of skin damage.

### 2.3 Selecting the studies

To compile the literature, EndNote X9 (Clarivate, London, UK) was utilised. The search results from the chosen databases were downloaded. EndNote X9 was used to screen, identify, and eliminate duplicate entries, followed by manually reviewing the list. R.V.M. and A.R.G. reviewed the article titles and abstracts to identify relevant studies. Articles that met the exclusion criteria were promptly removed. The full text of chosen articles was obtained and reviewed to ensure adherence to the inclusion criteria. In cases of disagreement between reviewers, S.F.M. and D.F.B. were consulted to provide an independent assessment and the final decision was made based on mutual agreement among all reviewers.

### 2.4 Charting the data

R.V.M. extracted the relevant data, such as authorship, year of publication, study title, study design, objectives, subject characteristics and housing conditions, source of UV, study intervention or exposure, treatment compound used, justification of pathway investigated, markers or antibodies used and key findings (results and discussion, conclusion, suggestions or limitations) from the chosen studies, using a standardised table.

### 2.5 Reporting the results

The scoping review aims to ascertain the effects of resveratrol and pterostilbene on skin photoaging models. The heterogeneous nature of the studies sparked an avenue to describe the search results and variables of interest that had been conducted. The study designs and major findings were summarised and presented in the review. Research gaps and nuances in skin photoaging models across studies were also explored.

## 3 Results

**Fig. 1:**
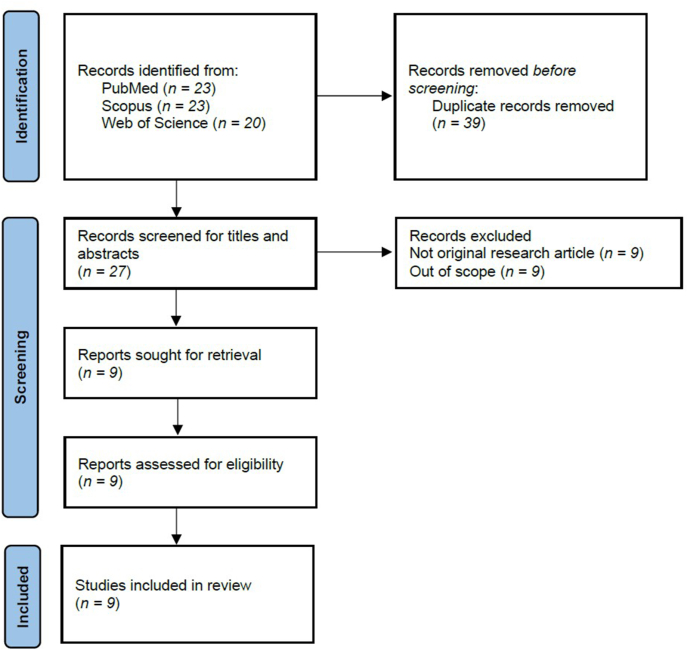
PRISMA flow chart for article search, screening and identification.

The literature search yielded 23 results in PubMed, 23 in Scopus, and 20 in Web of Science. Following the removal of duplicates, 27 unique articles were identified and subjected to screening. Then, 9 articles were excluded due to article type, and 9 were excluded due to the studies being out of scope. Subsequently, the full text of 9 articles was assessed. Ultimately, the review included 9 original research articles for analysis (Fig. 1).

The nine selected studies encompassed different study types: in vitro (n = 2), in vivo (n = 3) and a combination of in vitro and in vivo (n = 4). Predominantly, resveratrol was the stilbene of interest, with 8 out of 9 studies testing resveratrol against skin photoaging.

For the in vitro studies, Zhou et al. (2018) explored the effects of resveratrol on UVB-induced photoaging and apoptosis in HaCaT cells. UVB-induced cells pretreated with resveratrol exhibited a significant increase in cell viability. Next, resveratrol inhibited apoptosis by increasing expression of heat shock protein 27 (HSP27), decreasing expression of p-p65, Bax and cleaved caspase-3 (proapoptotic proteins) and increasing expression of Bcl-2 (anti-apoptotic protein). When the HSP27 gene was silenced, the responses were reversed, whereby resveratrol increased p-p65, Bax and cleaved caspase-3 and reduced Bcl-2. Hence, the study showed the anti-photoaging effects of resveratrol via an increase of HSP27 and inhibition of the apoptosis pathway, but further investigations using in vivo models are still warranted [42].

Next, Li et al. (2017) investigated pterostilbene and UVB-induced photodamage in HaCaT, specifically on the nuclear factor erythroid 2–related factor 2/antioxidant response element (Nrf2/ARE) pathway activation. Pterostilbene significantly prevented UVB-induced reduction in cell viability and increased reactive oxygen species (ROS) production, but the protective effect of pterostilbene was abolished by LY294002 (PI3K inhibitor) and Nrf2 silencing. Moreover, pterostilbene significantly increased gene expression of catalase (CAT), glutamate-cysteine ligase catalytic subunit (GCLC), glutamate-cysteine ligase modifier subunit (GCLM), glutathione reductase (GSR), heme oxygenase-1 (HMOX-1) and NAD(P)H quinone dehydrogenase 1 (NQO1); however, the trend was reversed for pterostilbene and LY294002 treatment. As for DNA damage, pterostilbene significantly diminished the UVB-induced DNA comet tail, but the comet tail was significantly increased when treated with pterostilbene and LY294002. Therefore, pterostilbene exhibited its photoprotective effects via activating the phosphoinositide 3-kinase (PI3K) pathway, which promoted Nrf2 nuclear translocation and induced antioxidant expression [43].

As for the in vivo studies, Arora et al. (2020) evaluated a novel vesicular formulation of resveratrol for anti-photoaging and antioxidant benefits. Female Swiss Albino mice aged 4–6 weeks old were treated with topical saline, resveratrol in conventional hydrogel or resveratrol loaded in transethosomal hydrogel, post-UV exposure for 4 weeks. Visual skin grading showed that both resveratrol groups significantly improved skin grade compared to control. As for the pinch test, the transethosomal group maintained extracellular matrix (ECM) protein similar to the control, while the conventional group did not reduce sagging time. Histologically, the transethosomal group showed maintenance of the epidermal layer, decreased hair follicles, minimal inflammation and normal elasto-collagen meshwork. The transethosomal group also raised superoxide dismutase (SOD), CAT, glutathione peroxidase (GSH-Px) and protein, with reduced malondialdehyde (MDA), while the conventional group showed opposite results. Therefore, resveratrol encapsulated in transethosomal vesicles bypassed its poor solubility and skin permeability [25].

In addition, Mostafa et al. (2021) investigated the effects of resveratrol and co-enzyme-Q10 towards UVA-induced skin photoaging in mice, emphasising the role of autophagy. Female CD1 mice were subjected to UVA-induced photoaging and daily oral resveratrol, co-enzyme-Q10 or control for 10 weeks. The resveratrol-treated photoaged mice exhibited a significant moderate reduction of deep wrinkles, decreased time in pinch test and increased glutathione (GSH). Histologically, resveratrol markedly improved epidermal atrophy, dermal inflammation and telangiectasia. Next, resveratrol demonstrated a complex modulation of autophagy: it decreased autophagy and increased inefficient autophagic flux in keratinocytes while increasing autophagy of inflammatory cells and promoting efficient autophagic flux in fibroblasts and inflammatory cells. Therefore, resveratrol showed protection against photoaging, but more studies need to detail the mechanism of resveratrol modulating autophagy under UVA-induced conditions [26].

Sirerol et al. (2015) used 10–12 weeks old SKH-1 hairless female mice, which were irradiated with an acute exposure of UVB before or 20 minutes after topical application of liposomes containing pterostilbene or resveratrol. Compared to after irradiation, the protective effects were significantly improved when stilbenes were applied prior to UVB irradiation. Hence, in subsequent experiments, mice were treated before irradiation. Pterostilbene prevented UVB-induced increases in skin fold, overall skin thickness, and skin redness better than resveratrol. Histologically, parameters related to photoaging, such as skin wrinkling and epidermal hyperplasia, were absent in pterostilbene-treated mice. As for transepidermal water loss (TEWL), which reflects skin barrier function, it was significantly increased in UVB-induced mice skin compared to control untreated mice. Mice treated with pterostilbene, or resveratrol showed similar TEWL values, that were not significant to control untreated mice. The authors concluded that pterostilbene prevents acute UVB-induced inflammation and photoaging better than resveratrol, and a highly extensive comparison between both stilbenes is still needed [29].

Most studies incorporated both in vitro and in vivo approaches. Cui et al. (2022) investigated the mechanism of resveratrol photoprotective properties in UVB-induced photoaging using HaCaT cells and 5-6 weeks-old male ICR mice. The in vitro results showed that pretreatment of resveratrol increased cell viability of UVB-irradiated cells, significantly reduced ROS, matrix metalloproteinase-1 (MMP-1), matrix metalloproteinase-9 (MMP-9), caspase3, caspase9, interleukin-6 (IL-6), tumor necrosis factor alpha (TNF-α), phosphorylated extracellular signal-regulated kinase 1/2 (P-ERK1/2) and phosphorylated p38 mitogen-activated protein kinase (P–P38MAPK), while significantly increasing glutathione disulfide (GSSH), SOD, nuclear Nrf2, glutathione peroxidase-4 (GPX-4), heme oxygenase-1 (HO-1) and vascular endothelial growth factor-B (VEGF-B). As for in vivo, mice treated orally with resveratrol showed similar skin surface as the control group with increased skin water content. Histologically, resveratrol improved hyperkeratosis, inflammatory cell infiltration, dermal collagen fibre density and type III collagen loss. Resveratrol also significantly decreased MMP-3, MMP-9, caspase3, caspase9, IL-6, phosphorylated c-Jun N-terminal kinase (P-JNK), P-ERK1/2, P–P38MAPK, while significantly increasing Nrf2, HO-1, NQO1, SOD1, and GPX-4. Thus, resveratrol does exhibit photoprotective effects against UVB-induced skin aging [27].

Kim et al. (2019) explored the effects of grape peel extract (GPE) and resveratrol on UVB-induced formation of skin wrinkles using HaCaT cells and 6-week-old male ICR mice. The in vitro results showed that resveratrol pretreatment for 24 hours, followed by UVB exposure, resulted in a slight increase of HO-1 and a significant increase of Nrf2. As for in vivo, resveratrol ameliorated UVB-induced wrinkle formation by decreasing average wrinkle number and total wrinkle length. In the skin and liver, nuclear Nrf2 and HO-1 were significantly increased dose-dependently with resveratrol, which was better than GPE. Resveratrol also significantly decreased MMP-1 and MMP-9 dose-dependently. Epidermal thickness was also reduced by about 55 % with resveratrol treatment. Thus, the authors posited that RES exerts its anti-wrinkle effects by activation of the Nrf2 signalling pathway [28].

Additionally, Lee et al. (2010) used human dermal fibroblasts (hDF) and 7–8 weeks old female adult albino hairless mice to study the effects of resveratrol on Sirtuin 1 (Sirt1) activation towards UVB-induced skin. In both UVB-induced hDFs and mice, resveratrol significantly decreased MMP-9 and maintained collagen IV levels. Metformin (Sirt1 agonist) also showed similar results to resveratrol. This is one of the earlier studies that showed resveratrol to be a Sirt1 agonist, making resveratrol a potential candidate to minimise or impede photoaging through inhibition of collagen degradation in UV-induced skin alongside metformin [44].

Finally, Xia et al. (2024) investigated resveratrol's anti-aging mechanism via the AMP-activated protein kinase (AMPK) pathway using human skin fibroblasts (HSF) and 6 weeks old male BALB/c mice. Resveratrol pretreatment on UVA-induced HSFs improved cell morphology and viability, suppressed apoptosis, restored normal cell cycle progression, reduced ROS production, slowed aging and promoted autophagy via the AMPK pathway. Similarly, in vivo results showed that resveratrol mitigated photoaging through AMPK-activated autophagy. The role of the AMPK pathway was further confirmed using autophagy and AMPK inhibitors. Moreover, resveratrol enhanced the appearance of mice skin at both macroscopic and histological levels. Overall, resveratrol improves photoaging, while autophagy inhibition worsens photoaging [45].

The studies included are summarised in Table 1. Overall, these studies highlight the various effects of pterostilbene and resveratrol on skin photoaging across in vitro and in vivo models, with a strong emphasis on the development of photoaging models and the parameters investigated. These findings underscore the need to link what has been discovered thus far, to better define the potential of pterostilbene and resveratrol as anti-photoaging agents or cosmeceuticals.

**Table 1:** Effects of pterostilbene and resveratrol on skin photoaging.

Study	Study Design	Major Findings
Zhou et al., 2018 [42]	***Type of study:*** In vitro ***Stilbene of interest:*** Resveratrol ***Cell:*** HaCaT cells ***Type of UV irradiation:*** UVB ***UV dose exposure plan:*** Cells were irradiated for 5 min, d = 10 cm, with an irradiation intensity of 0.1 mW/cm^2^. The irradiation dose was 30 mJ/cm^2^. ***Groups*** (1) ***Control:*** cells that were not irradiated (2) ***Model:*** UVB irradiated cells (3) ***Resveratrol:*** UVB irradiated cells with pretreatment of 2.5, 5, 7.5, and 10 μM resveratrol (dissolved in DMSO) ***Positive control:*** No	***CCK8 cell viability assay:*** UVB-induced HaCaT cells treated with resveratrol showed significantly ↑ viability dose-dependently.
		***Apoptotic rate:*** Apoptotic rate of UVB-induced cells was 35.01 %, while UVB-induced cells with pretreatment of 5 and 10 μM resveratrol ↓ to 20.12 % and 12.59 %, showing that resveratrol significantly ↑ cell viability.
		***Relationship between HSP27 and apoptosis pathway:*** Resveratrol inhibited apoptosis by ↑ expression of HSP27, ↓ expression of p-p65, Bax, and cleaved caspase-3 and also ↑ expression of Bcl-2.
		After HSP27 gene silencing, UVB-induced cells with pretreatment of resveratrol led to ↑ Bax, ↓ Bcl-2, and ↑ p-p65 and caspase-3 activation.
Li et al., 2017 [43]	***Type of study:*** In vitro ***Stilbene of interest:*** Pterostilbene ***Cell:*** HaCaT cells ***Type of UV irradiation:*** UVB ***UV dose exposure plan:*** Cells were irradiated with 300 mJ/cm^2^. ***Groups*** (1) ***Control:*** No UVB exposure and no treatment (2) ***Model:*** UVB exposure with no treatment (3) ***Pterostilbene (Pter):*** Pretreatment of 5 and 10 μM Pter for 24 h prior to UVB or no UVB irradiation. Pter was prepared in DMSO. ***Positive control:*** No	***MTT assay:*** 5 and 10 μM Pter exhibited no noticeable effect towards cell viability. Pter significantly prevented UVB-induced ↓ in cell viability, whereas when Nrf2 was silenced, Pter's protective effect was abolished.
		***PI3K pathway:*** LY294002 (PI3K inhibitor) significantly ↓ the cell viability of UVB-induced HaCaT cells, completely diminishing Pter (5 μM) protective effects.
		Pter activates the PI3K signalling pathway, which induces Nrf2 phosphorylation and translocation, but without altering Keap1 protein expression.
		***Antioxidant pathway:*** Pretreatment of Pter prior to UVB irradiation significantly ↓ ROS generation. However, LY294002 eliminated the inhibition of ROS production, mediated by Pter.
		Pter treatment significantly ↑ mRNA expression of CAT, GCLC, GCLM, GSR, HMOX-1, and NQO1. However, Pter + LY294002 reversed this trend and ↓ the gene expression.
		***DNA damage:*** Pter or LY294002 on their own showed no effect on DNA. Pretreatment of Pter significantly ↓ UVB-induced DNA comet tail compared to UV-irradiated control (which was significantly ↑). However, DNA repair activity induced by Pter (due to UVB) was effectively inhibited by LY294002, which significantly ↑ DNA comet tail.
Arora et al., 2020 [25]	***Type of study:*** In vivo ***Stilbene of interest:*** Resveratrol ***Animal:*** 4–6 weeks old 24 female Swiss Albino mice ***Type of UV irradiation:*** UV simulating the full solar spectrum ***UV dose exposure plan:*** Mice were anesthetised to restrict movement, thus providing uniform and homogenous UV exposure. UV exposure was carried out once a day, 5 times a week. Mice were irradiated at d = 40 cm for 5 min. ***Method of shaving:*** Ketamine i.m. (40 mg/kg) anesthesia for shaving of mice dorsal skin (2 × 2 cm^2^) ***Number of mice per group:****n* = 6/group ***Groups*** (1) ***Group 1:*** Control, topical saline with no UV exposure (2) ***Group 2:*** Topical saline with UV exposure (3) ***Group 3:*** UV exposure, followed by conventional resveratrol hydrogel (equivalent to 25 mg resveratrol) post-exposure (4) ***Group 4:*** UV exposure, followed by resveratrol-loaded transethosomal hydrogel formulation (equivalent to 25 mg resveratrol) post-exposure. ***Positive control:*** No ***Period of study:*** 4 weeks	***Visual skin grading:*** Group 2 showed significantly prominent skin wrinkles, while Group 3 and 4 showed significantly improved skin grade compared to control.
		***Pinch test:*** Group 4 maintained ECM protein similar to the control, while Group 3 did not reduce sagging time compared to the control.
		***Histopathological findings:*** Via H&E staining, Group 2 showed collagen bundle destruction, epidermal inflammation and damage, and also solar elastosis, similar to Group 3. Group 4 showed maintenance of epidermal layer integrity, decreased hair follicles and negligible inflammation on the skin's upper portion and normal elasto-collagen meshwork.
		***Biomarkers of skin aging:*** Groups 2 and 3 showed ↓ SOD, CAT, GSH-Px and protein while ↑ MDA compared to control. Group 4 showed ↑ SOD, CAT, GSH-Px and protein while ↓ MDA compared to Group 2, but similar to control.
Mostafa et al., 2021 [26]	***Type of study:*** In vivo ***Stilbene of interest:*** Resveratrol ***Animal:*** 60 female CD1 mice ***Type of UV irradiation:*** UVA ***UV dose exposure plan:*** Mice in photoaged groups were exposed to UVA 3 times a week with increasing doses. The first dose was 1 minimal erythema dose (MED) = 80 mJ/cm^2^ in Week 1, increasing 1 MED per week to reach 4 MED in Week 4, which was then maintained at 4 MED until Week 10. Mice in normal control group were exposed to white light. ***Method of shaving:*** About 2.5 × 3 cm of mice dorsal skin were shaved clean ***Number of mice per group:****n = 12/group* ***Groups*** (1) ***Group 1:*** Normal control - gum acacia. Treatment was administered once a day via oral gavage for 10 weeks. (2) ***Group 2:*** Untreated photoaged - gum acacia (3) ***Group 3:*** Resveratrol (50 mg/kg) photoaged (4) ***Group 4:*** Co-enzyme-Q10 (100 mg/kg) photoaged (5) ***Group 5:*** Resveratrol (50 mg/kg) + co-enzyme-Q10 (100 mg/kg) photoaged ***Positive control:*** No ***Period of study:*** 11 weeks (including 1 week of acclimatisation)	***Macroscopic evaluation:*** Group 2 showed deep wrinkles, which were significantly moderately attenuated in Group 3.
		***Pinch test:*** Group 3 showed significantly ↓ time.
		***Oxidative stress:*** Group 3 significantly ↑ GSH and not significant for MDA.
		***H&E and scoring:*** Group 2 showed epidermal thinning, elastic and collagen fibre fragmentation, telangiectasia, and excessive inflammatory cell infiltration in the dermis. Marked improvement of epidermal atrophy, dermal inflammation and telangiectasia were observed in Group 3.
		***Autophagy markers:*** Via IHC, Group 2 showed significant ↑ of LC3II positively stained keratinocytes, fibroblasts and inflammatory cells. Group 3 showed a significant ↓ in positively stained keratinocytes, not significant in fibroblasts and significant ↑ in inflammatory cells, compared to Group 2.
		Group 2 showed a mild significant ↑ of P62-positive keratinocytes, which was significantly ↑ in Group 3. While a significant ↑ of P62-positive fibroblast and inflammatory cells were detected in Group 2, it was significantly ↓ in Group 3.
Sirerol et al., 2015 [29]	***Type of study:*** In vivo ***Stilbene of interest:*** Pterostilbene and Resveratrol ***Animal:*** 40 female 10–12 weeks old SKH-1 hairless mice ***Type of UV irradiation:*** UVB ***UV dose exposure plan:*** Mice were irradiated once with 360 mJ/cm^2^ (2 times the UVB MED in albino hairless mice) before or 20 min after topical treatment ***Method of shaving:*** Not applicable ***Number of mice per group:****n = 10/group* ***Groups*** (1) ***Control untreated:*** No UVB exposure and no vehicle (2) ***UVB:*** UVB exposure and no vehicle (3) ***Vehicle + UVB:*** UVB exposure and topical application of liposomes (containing lecithin) diluted in Carbopol, 1:1 ratio (4) ***Pterostilbene (Pter) + UVB:*** UVB exposure and topical application of liposomes (containing 20 μM Pter) diluted in Carbopol, 1:1 ratio. The final concentration was 10 μmol/200 μL (5) ***Resveratrol (Resv) + UVB:*** UVB exposure and topical application of liposomes (containing 20 μM Resv) diluted in Carbopol, 1:1 ratio. The final concentration was 10 μmol/200 μL ***Positive control:*** No ***Period of study:*** < 1 week	***Pre- or post-UVB exposure for treatment application:*** Protective effects were significantly better when stilbenes were applied before rather than after UVB irradiation. For further experiments, treatment was applied to mice before UVB. ***Skin fold:*** 24 h after UVB irradiation, Pter prevented ↑ in skin fold, overall skin thickness, and skin redness, better than prevention by Resv. ***H&E staining:*** With UVB irradiation, skin wrinkling and epidermal hyperplasia (classical parameters related to photoaging) were more evident. Pter mice with UVB showed absence of hyperplasia and inflammatory cell infiltrate. ***TEWL:*** TEWL was ↑ significantly in UVB-induced mice skin (24 h after UVB) compared to control untreated mice. As for UVB-induced mice treated with Pter or Resv, their TEWL values were similar (not significant) to control untreated mice.

Cui et al., 2022 [27]	***Type of study:*** In vitro and in vivo ***Stilbene of interest:*** Resveratrol ***Cell:*** HaCaT cells ***Type of UV irradiation:*** UVB ***UV dose exposure plan:*** Cells (covered by PBS) were irradiated with 50 mJ/cm^2^ **Groups** (1) ***Control:*** No UVB exposure and no treatment (2) ***Model:*** UVB exposure with no treatment (3) ***Group 3–6:*** UVB exposure with resveratrol pretreatment at concentrations of 10, 20, 40, and 60 μM ***Positive control:*** No ***Animal:*** 5–6 weeks old 30 male ICR mice ***Type of UV irradiation:*** UVB ***UV dose exposure plan:*** Mice were irradiated 3 times a week. 40 mJ/cm^2^ (Week 4), 80 mJ/cm^2^ (Week 5), 120 mJ/cm^2^ (Week 6) and 120 mJ/cm^2^ (Week 7) ***Method of shaving:*** Mice back were shaved with the use of electric razor and hair removal cream ***Number of mice per group:****n = 10/group* ***Groups*** (1) ***Control:*** Oral saline with no UVB exposure (2) ***Model:*** Oral saline with UVB exposure (3) ***Treatment:*** Oral gavage of 2 mg/kg resveratrol (dissolved in ultrapure water with 10 % ethanol and 10 % Tween 80) with UVB exposure. Treatment started from Week 2, 3 times a week, 30 min prior to each irradiation ***Positive control:*** No ***Period of study:*** 7 weeks	***In vitro***
		***MTT assay:*** Resveratrol at 10, 20, 40, and 60 μM ↑ cell viability of UVB irradiated cells.
		***Anti-MMP expression:*** Resveratrol significantly ↓ MMP-1 and MMP-9.
		***Anti-apoptosis:*** Resveratrol significantly ↓ caspase3 and caspase9 compared to the model group. On mRNA level, resveratrol did not change caspase3.
		***Antioxidant:*** Resveratrol showed significant ↑ in GSSH, SOD, nuclear Nrf2, GPX-4 and HO-1, with significant ↓ in ROS.
		***Anti-inflammatory:*** Resveratrol significantly ↓ IL-6 and TNF-α.
		***COX-2 and MAPK pathway-related proteins:*** Resveratrol significantly ↓P-ERK1/2 and P–P38MAPK only. No effect was seen for ERK1/2 and P38MAPK.
		***VEGF-B expression:*** Resveratrol significantly ↑ VEGF-B mRNA expression.
		***In vivo***
		***Skin image analysis:*** The model group showed rough and hypertrophic skin, with erythema, peeling, ulceration, and deep wrinkles. The treatment group skin surface was similar to the control group. The treatment group also showed significant ↑ in skin water content.
		***Histopathological findings:*** For H&E staining, the model group showed ↑ hyperkeratosis, ↑ inflammatory cell infiltration, and dermal collagen fibres in scattered and fractured arrangement. These conditions were improved in the treatment group.
		For MT staining, the model group had a significant ↓ in blue-stained collagen fibres in the dermis. The treatment group countered the ↓ in collagen fibres significantly.
		For IHC, the treatment group alleviated UVB-induced collagen loss in mice skin, shown by significantly ↑ type III collagen immunoreactivity.
		***Anti-MMP expression:*** MMP-3 and MMP-9 were significantly ↓ in the treatment group.
		***Anti-apoptosis:*** Caspase3 and caspase9 were significantly ↓ in the treatment group.
		***Antioxidant:*** Nrf2, HO-1, NQO1, SOD1, and GPX-4 (especially NQO1) significantly ↑ in treatment group.
		***Anti-inflammatory:*** IL-6 significantly ↓ in the treatment group.
		***COX-2 and MAPK pathway-related proteins:*** P-JNK, P-ERK1/2, P–P38MAPK expression were significantly ↓ in the treatment group.
Kim et al., 2019 [28]	***Type of study:*** In vitro and in vivo ***Stilbene of interest:*** Resveratrol ***Cell:*** HaCaT cells ***Type of UV irradiation:*** UVB ***UV dose exposure plan:*** Cells were irradiated with 25 mJ/cm^2^ **Groups** (1) ***Control:*** No UVB exposure and no treatment (2) ***Model:*** UVB exposure with no treatment (3) ***Grape peel extract (GPE):*** UVB or no UVB exposure with GPE at concentrations of 156, 313 and 625 μg/ml (4) ***Resveratrol (RES):*** UVB or no UVB exposure with RES at concentrations of 2 and 8 μM ***Positive control:*** No ***Animal:*** 6 weeks old 56 male ICR mice ***Type of UV irradiation:*** UVB ***UV dose exposure plan:*** Mice were irradiated 3 times per week for 4 weeks. 40 mJ/cm^2^ (Week 1), 80 mJ/cm^2^ (Week 2), 120 mJ/cm^2^ (Week 3 and 4) ***Method of shaving:*** Mice back were shaved and applied with waxing cream ***Number of mice per group:****n = 8/group* ***Groups*** (1) ***Group 1:*** No UVB exposure (2) ***Group 2:*** Oral gavage of vehicle (drinking water with 10 % v/v ethanol and 10 % v/v Tween-80) and UVB exposure (3) ***Group 3:*** Oral gavage of RES (2 mg/kg bw) and UVB exposure. RES was dissolved in vehicle (4) ***Group 4:*** Oral gavage of RES (10 mg/kg bw) and UVB exposure (5) ***Group 5:*** Oral gavage of RES (50 mg/kg bw) and UVB exposure (6) ***Group 6:*** Oral gavage of GPE (1000 mg/kg bw) and UVB exposure. GPE was dissolved in vehicle (7) ***Group 7:*** Oral gavage of GPE (2000 mg/kg bw) and UVB exposure. Treatment started from the 2nd week, 3 times per week, for 2 weeks. For the next 4 weeks, mice were fed 3 times a week, 1 h before UVB exposure. ***Positive control:*** No ***Period of study:*** 7 weeks (including 1 week of acclimatisation)	***In vitro***
		***Antioxidant:*** Pretreatment of RES for 24 h, followed by UVB exposure, resulted in slightly ↑ HO-1 and significantly ↑ Nrf2.
		***In vivo***
		***Anti-wrinkle effect:*** UVB exposure resulted in ↑ of total wrinkle area. RES tend to ameliorate UVB-induced wrinkle formation. The average number of wrinkles was ↓ by all RES concentrations, especially at 50 mg/kg bw. The total wrinkle length (mm) was most ↓ at 10 mg/kg bw RES.
		***Antioxidant on mice skin and liver:*** The vehicle group (Group 2) showed significantly ↓ nuclear Nrf2 and HO-1 while RES groups resulted in a significant ↑ dose dependently, with the most ↑ at 50 mg/kg bw, better than GPE.
		***MMPs:*** MMP-1 ↑ in the vehicle, but with RES groups there was a dose-dependent ↓, with the most ↓ at 50 mg/kg bw. MMP-9 was also ↑ in the vehicle, while RES treatment ↓ MMP-9 significantly.
		***Histological analysis:*** Epidermal thickness was significantly ↑ in the vehicle, with a moderate inflammatory cell infiltration in the dermis. With RES treatment, the epidermal thickness ↓ by about 55 %.
Lee et al., 2010 [44]	***Type of study:*** In vitro and in vivo ***Stilbene of interest:*** Resveratrol ***Cell:*** Human dermal fibroblast (hDF) isolated from human skin samples ***Type of UV irradiation:*** UVB ***UV dose exposure plan:*** Cells were irradiated with 30 mJ/cm^2^. ***Groups*** (1) ***Control:*** No UVB exposure, no pre- and post-treatment (2) ***Model:*** UVB exposure with no pre- and post-treatment (3) ***Resveratrol:*** Pretreatment of resveratrol at 50 μM for 1 h, followed by UVB exposure and post-treatment of resveratrol for 24 h (4) ***Metformin (Sirt1 agonist):*** Pretreatment of metformin at 5 mM for 1 h, followed by UVB exposure and post-treatment of metformin for 24 h ***Positive control:*** No ***Animal:*** 7–8 weeks old 10 female adult albino hairless mice ***Type of UV irradiation:*** UVB ***UV dose exposure plan:*** Mice were irradiated once on day 2 at 180 mJ/cm^2^ ***Method of shaving:*** Not applicable ***Number of mice per group:****n = 2/group* ***Groups*** (1) ***Group 1:*** Control – no treatment and no UVB exposure (2) ***Group 2:*** Vehicle only - 200 μl PBS was injected intradermally to the dorsal area with a 30G needle syringe 24 h prior to UVB exposure (3) ***Group 3:*** Resveratrol (5 mM) injected intradermally (4) ***Group 4:*** Metformin (5 mM) injected intradermally (5) ***Group 5:*** Metformin (10 mM) injected intradermally ***Positive control:*** No ***Period of study:*** 3 days (day 1 for treatment, day 2 for UVB exposure and day 3 for culling)	***In vitro***
		Resveratrol and metformin significantly ↓ UV-induced MMP-9.
		Collagen IV levels of UV-induced hDFs were maintained in resveratrol and metformin-treated groups, consistent with ↓ MMP-9.
		***In vivo***
		Post-UVB exposure revealed that MMP-9 at mRNA, protein and enzyme activity levels were significantly blocked with resveratrol & metformin pretreatment.
		Collagen IV levels of UVB-induced mice skin with resveratrol pretreatment appear comparable to the control group.
Xia et al., 2024 [45]	***Type of study:*** In vitro and in vivo ***Stilbene of interest:*** Resveratrol (RSV) ***Cell:*** Human skin fibroblasts (HSF) ***Type of UV irradiation:*** UVA ***UV dose exposure plan:*** Cells were irradiated with 16 J/cm^2^ **Groups** (1) ***Control:*** No UVA exposure, no treatment (2) ***UVA:*** UVA exposure with no treatment (3) ***UVA + RSV:*** Pretreatment of RSV (5, 10, 25, 50, 75, 100 and 200 μM) followed by UVA exposure (4) ***UVA +* 3-MA*:*** Pretreatment of 3-MA (3-methyladenine; autophagy inhibitor) followed by UVA exposure ***Positive control:*** No ***Animal:*** 6 weeks old 30 male BALB/c mice ***Type of UV irradiation:*** UVA ***UV dose exposure plan:*** In the UVA group, mice were coated with 8-MOP (8-methoxy psoralen) on their backs and irradiated for 8 weeks. MED of 0.35 J/cm^2^ UVA was given, and the dose escalated by 5 % daily for 8 weeks. If the mice skin showed signs of erythema or inflammation on the next day post-exposure, mice were rested for 2 days, and the previous dose was continued, with a one day pause after 4 consecutive days of exposure. ***Method of shaving:*** Mice were placed in a gas anesthesia machine to facilitate back hair removal. The exact method of hair removal was not mentioned. ***Number of mice per group:****n = 6/group* ***Groups*** (1) ***Control:*** No UVA exposure, no treatment (2) ***UVA:*** UVA exposure, no treatment (3) ***UVA + RSV:*** UVA exposure with 100 μmol/L RSV injected s.c. (4) ***UVA +* 3-MA*:*** UVA exposure with 3-MA injected s.c. (5) ***UVA + PBS:*** UVA exposure with PBS injected s.c. Treatment started after 8 weeks of UVA irradiation. RSV, 3-MA and PBS were administered 2 times a week for the next 8 weeks. ***Positive control:*** No ***Period of study:*** 17 weeks (including 1 week of acclimatisation)	***In vitro***
		***Cell morphology:*** HSF showed spindle-shaped morphology, but UVA-irradiated HSF showed severe atrophy with irregular morphology and ↑ cellular debris. HSF pretreated with RSV followed by UVA irradiation, ↓ cell morphology and ↓ cellular debris.
		***CCK8 cell viability analysis:***
		Pretreatment of HSF with 5–200 μM RSV, followed by UVA exposure, showed 81.17 % cell viability (100 μM RSV) and 57.03 % cell viability (200 μM RSV).
		***Annexin V-FITC for apoptosis:***
		UVA showed ↑ early apoptosis rate (0.33 % to 36.8 %) and ↑ late apoptosis rate (1.90 % to 11.1 %) compared to the control.
		UVA + 3-MA slightly ↑ early apoptosis rate and further ↑ late apoptosis rate, from 11.1 % to 19.3 %, compared to the UVA group.
		UVA + RSV significantly ↓ early apoptosis rate (dropped to 5.04 %), which indicates apoptosis inhibition.

		***PI staining for cell cycle analysis:***
		UVA significantly caused ↑ G1-phase arrest of HSF, from 43.0 % to 82.8 %) when compared to control. UVA + RSV ↓ G1-phase arrest of HSF to 58.7 %, restoring normal cell cycle.
		***Oxidative stress assay, DCFH-DA:***
		UVA showed ↑ ROS, while UVA + 3-MA showed further ↑ ROS, proving that inhibition of autophagy increases ROS levels. UVA + RSV ↓ ROS dose-dependently, which indicates that RSV promotes autophagy to ↓ ROS production.
		***SA-β-gal staining:***
		UVA showed ↑ SA-β-gal activity of HSF, while UVA + RSV significantly ↓ SA-β-gal activity and UVA + 3-MA ↑ SA-β-gal activity. This indicates that RSV induces autophagy to impede the aging process, while autophagy inhibition accelerates aging.
		***Collagen I and MMP1:*** UVA ↓ collagen I and ↑ MMP1 expression, while UVA + RSV ↑ collagen I and ↓ MMP1 expression.

		***Autophagy markers:*** UVA showed ↑ p62, ↓ Beclin-1 and ↓ LC3B, while UVA + RSV or RSV only ↓ p62, ↑ Beclin-1 and ↑ LC3B, indicating RSV promotes autophagy in HSF.

		***AMPK inhibition:*** AMPK inhibitor Compound C (CC) was also added. UVA + RSV + CC ↓ AMPK and *p*-AMPK expression levels, while UVA + RSV ↑ AMPK phosphorylation, which ↑ *p*-AMPK/AMPK expression levels.
		UVA + RSV + CC further ↑ p62, ↓ Beclin-1 and ↓ LC3B.
		UVA + RSV ↓ p21, while UVA ↑ p21 and UVA + RSV + CC ↑ p21. This shows that RSV can activate autophagy to improve UVA-induced photoaging via the AMPK signalling pathway.
		***Immunofluorescence:*** UVA + RSV showed ↑ LC3B, while UVA showed ↓ LC3B.
		***In vivo***
		***Skin macroscopic appearance:***
		UVA showed ↑ erythema, wrinkles and hyperpigmentation after 8 weeks. UVA + RSV showed ↓ erythema and wrinkles, while UVA + 3-MA worsened photoaging compared to UVA + PBS.
		***Histological analysis:***
		The control group showed a well-organised epidermis and a distinct epidermal-dermal demarcation. The wavy fibrous tissues in the dermis were distributed uniformly and arranged orderly, without inflammatory cell infiltration.
		UVA group showed hyperplastic epidermis with hyperkeratosis. Collagen fibres in the dermis were degraded, damaged, decreased, disorganised and unevenly distributed, with inflammatory cell infiltration.
		UVA + 3-MA showed ↑ epidermal thickening and ↓ collagen fibres with a significantly ↑ inflammatory cell infiltration compared to UVA + PBS, demonstrating that autophagy inhibition increased photoaging.
		UVA + RSV showed dramatically ↓ hyperkeratosis, ↓ epidermis thickness, ↑ collagen fibre with a considerable ↓ in inflammatory cell infiltration and necrotic shedding of epidermal cells.
		***Autophagy and cell cycle markers:***
		UVA showed ↓ Beclin-1, ↓ LC3B, ↑ p62 and ↑ p21, while UVA + RSV showed ↑ Beclin-1, ↑ LC3B, ↓ p62 and ↓ p21.

		***AMPK signalling:*** UVA showed significantly ↓ *p*-AMPK/AMPK expression, while UVA + RSV significantly ↑ *p*-AMPK/AMPK expression.
		***MMP1 expression:*** UVA showed significantly ↑ MMP1, while UVA + RSV significantly ↓ MMP1.

Notes: ↑: increase; ↓: decrease. Abbreviations: UV: ultraviolet; CCK8: Cell Counting Kit-8; HSP27: heat shock protein 27; p-: phosphorylated; Bax: Bcl-2-associated X protein; Bcl-2: B-cell lymphoma 2; Nrf2: nuclear factor erythroid 2-related factor 2; PI3K: phosphoinositide 3-kinase; Keap1: Kelch-like ECH-associated protein 1; ROS: reactive oxygen species; CAT: catalase; GCLC: glutamate-cysteine ligase catalytic subunit; GCLM: glutamate-cysteine ligase modifier subunit; GSR: glutathione-disulfide reductase; HO-1/HMOX-1: heme oxygenase 1; NQO1: NAD(P)H quinone dehydrogenase 1; SOD: superoxide dismutase; GSH-Px/GPx: glutathione peroxidase; GSH: glutathione (reduced); MDA: malondialdehyde; ECM: extracellular matrix; MED: minimal erythema dose; H&E: hematoxylin and eosin staining; IHC: immunohistochemistry; LC3: microtubule-associated proteins 1A/1B-light chain 3; TEWL: transepidermal water loss; MTT: (3-[4,5-dimethylthiazol-2-yl]-2,5 diphenyl tetrazolium bromide); MMP: matrix metalloproteinase; GSSH: GSH/oxidised glutathione; COX-2: cyclooxygenase-2; TNF-α: tumor necrosis factor alpha; JNK: c-Jun N-terminal kinase; p38MAPK: p38 mitogen-activated protein kinases; ERK1/2: extracellular signal-regulated kinases 1/2; VEGF-B: vascular endothelial growth factor B; Sirt: sirtuin; MT: Masson's Trichrome staining; SA-β-gal: Senescence-associated beta-galactosidase; AMPK: AMP-activated protein kinase.

To further elucidate their photoprotective potential, it is essential to understand the mechanisms underlying their effects. Table 2 outlines the key mechanisms of action through which pterostilbene and resveratrol exert their photoprotective effects on skin photoaging.

**Table 2:** Mechanisms of action of pterostilbene and resveratrol on skin photoaging.

Stilbene	Mechanisms of action
Resveratrol (in vitro)	***Cell viability and morphology***
	• Enhanced cell viability [27,42]
	• Preserved cell viability, maintained normal cell shape and decreased cellular debris [45]
	***Molecular pathways involved***
	• Enhanced antioxidant defense [27,28]
	• Reduced ROS [27,45]
	• Reduced inflammation [27]
	• Inhibited MMPs: MMP-1 [27,45] and MMP-9 [27,44,45]
	• Increased collagen type I [45] and preserved collagen type IV [44]
	• Suppressed apoptosis [27,42,45]
	• HSP27 silencing reversed resveratrol's anti-apoptotic effects [42]
	• Activated autophagy and prevented excessive G1-phase arrest, supporting normal cell proliferation [45]
	• Modulated the COX-2 and MAPK pathways [27]
	•Enhanced AMPK phosphorylation [45]
	• Upregulated VEGF-B mRNA expression [27]
	• Exhibited anti-aging effects by reduction of SA-β-gal activity [45]
Resveratrol (in vivo)	***Skin macroscopic appearance***
	• Reduced wrinkles [[25], [26], [27], [28],^45^]
	• Reduced inflammation [25,26,45]
	• Decreased erythema and hyperpigmentation [45]
	***Histological changes***
	• Preserved epidermal integrity [25]
	• Reduced epidermal thinning [26]
	• Reduced epidermal thickness [28,45]
	• Reduced hyperkeratosis [27,45]
	• Prevented collagen degradation [25,26]
	• Restored collagen fibers [27,45]
	***Other structural and functional properties of skin***
	• Improved skin elasticity [26]
	• Maintained skin hydration [27,29]
	***Molecular pathways involved***
	• Enhanced antioxidant defense [[25], [26], [27], [28]]
	• Decreased MDA [25]
	• Reduced inflammation [27]
	• Inhibited MMPs: MMP-1 [28,45], MMP-3 [27] and MMP-9 [27,28,44]
	• Increased type III collagen [27] and preserved type IV collagen [44]
	• Regulated autophagy [26]
	• Enhanced AMPK phosphorylation, activated autophagy and regulated the cell cycle [45]
	• Modulated the COX-2 and MAPK pathway [27]
	• Suppressed apoptosis [27]
Pterostilbene (in vitro)	***Cell viability***
	• Protected against UVB-induced cell viability loss via PI3K/Nrf2 activation [43]
	***Molecular pathways involved***
	• Activated PI3K signaling, inducing Nrf2 phosphorylation and translocation without altering Keap1 [43]
	• Reduced ROS and enhanced antioxidant response [43]
	• Mitigated UVB-induced DNA damage, but this protection was lost with PI3K inhibition [43]
Pterostilbene (in vivo)	***Skin macroscopic appearance***
	• Prevented skin thickening, increased skin folds and redness more effectively than resveratrol [29]
	***Histological changes***
	• Reduced epidermal hyperplasia and inflammatory cell infiltration post-UVB [29]
	***Other structural and functional properties of skin***
	• Maintained skin hydration [29]

## 4 Discussion

Our findings showed that a majority of studies have used resveratrol as a stilbene of interest in treating skin photoaging. Regardless, the properties of resveratrol and pterostilbene are interconnected and can be utilised to effectively target and alleviate skin photoaging. Skin photoaging occurs due to the interplay between solar ultraviolet (UV) radiation, oxidative stress and collagen breakdown. When the skin is subjected to prolonged and repeated exposures to UV radiation, oxidative stress increases. The increased oxidative stress brings about inflammation and increases matrix metalloproteinases (MMPs) that breakdown collagen in the skin [2,46,47]. In turn, the skin shows photoaging characteristics such as coarse wrinkles, laxity and rough skin [9,10,48]. Although the skin has endogenous antioxidants, that too diminishes over time, especially with prolonged oxidative stress. To counter oxidative stress, we need exogenous forms of antioxidants, such as stilbenes that have shown promising results in ameliorating skin photoaging.

### 4.1 Stilbenes and in vitro studies

Firstly, more in vitro studies have been conducted on how resveratrol mitigates skin photoaging through multiple mechanisms. Zhou et al. (2018) found that resveratrol decreased apoptosis and inflammation by increased heat shock protein 27 (HSP27) gene expression, decreased expression of p-p65, Bax, and cleaved caspase-3, and also increased expression of Bcl-2 [42]. Next, Cui et al. (2022) found that resveratrol reduced apoptosis, inflammation and collagen breakdown while boosting antioxidant defenses [27]. Kim et al. (2019) and Lee et al. (2010) supported these findings by showing increases in nuclear factor erythroid 2-related factor 2 (Nrf2) and preservation of collagen, respectively [28,44]. Additionally, Xia et al. (2024) delved deeper into the autophagy mechanism and found that resveratrol promoted AMP-activated protein kinase (AMPK)-mediated autophagy, improving photoaging [45]. As for pterostilbene, there was only one in vitro study. Li et al. (2017) found that pterostilbene significantly prevented UVB-induced decrease in cell viability, reduced ROS generation, increased antioxidant gene expression, and reduced DNA damage. However, when phosphoinositide 3-kinase (PI3K) was inhibited, the effects of pterostilbene were diminished entirely. This implies that PI3K is vital in mediating pterostilbene's protective effects [43].

### 4.2 In vitro skin photoaging model considerations

There is no definitive set of guidelines when establishing an in vitro skin photoaging model. Thus, we reviewed trends across studies in terms of cell type, UV radiation type and the UV dose exposed to cells. Most studies used HaCaT cells [27,28,42,43], except for 2 studies using human dermal fibroblasts (hDF) isolated from human skin samples [44] and human skin fibroblasts (HSF) [45]. HaCaT cells are an immortalised human keratinocyte cell line used for many skin-related studies [49]. However, one study that compared HaCaT cells to normal human epidermal keratinocytes (NHEK) found that HaCaT cells showed dysregulated signal transduction, transcription and post-translation compared to NHEK. So, the study's authors do not recommend using HaCaT for pharmacological screening of novel photoprotective compounds to prevent incorrect predictions to human skin [50].

As for hDF and HSF, these are primary cells directly isolated from living tissues, such as through biopsies or surgical samples, and cultured for growth in vitro. Despite its finite life span, primary cells retain their original characteristics and functions [51], unlike immortalised cell lines, which are genetically manipulated and passaged countless times, leading to genotype and phenotype variations deviating from normal responses [51,52]. Despite this, cell lines are extensively utilised in research due to its cost-effectiveness, ease of use and ability to bypass ethical concerns. While practical, caution is necessary when interpreting results because cell lines do not always behave identically to primary cells. Therefore, if cell lines were used, findings should be verified using primary cells [52,53].

Ultimately, both cell lines and primary cells lack the local environment found in vivo, which may influence interactions with other cells that are crucial to the tested hypothesis [52]. As a result, we recommend that in vitro models be used alongside in vivo models to provide a more comprehensive assessment of potential photoprotective compounds.

As for the type of UV used, most studies investigating skin photoaging in vitro used UVB irradiation [27,28,[42], [43], [44]], with only one opting for UVA [45]. UVB was the choice of irradiation, probably due to its higher energy and 1000 times more erythema-causing than UVA [54], resulting in greater cellular damage via increased ROS production. Next, the dosage used to induce photoaging in an in vitro model presented a wide range. Most studies employed doses between 5 and 30 mJ/cm^2^ [27,28,42,44], while one study used a significantly higher dose of 300 mJ/cm^2^ [43] when UVB was used. For UVA, a dose of 16 J/cm^2^ (equivalent to 16,000 mJ/cm^2^) was used [45], indicating that a higher dose of UVA is needed to elicit effects compared to UVB. The variability in dosage likely comes from the necessity to optimise experimental conditions, with different cell lines and culture setups requiring tailored UV doses to induce photoaging.

Most in vitro studies from this review had pretreated cells with either resveratrol or pterostilbene with the exception of one study by Lee et al. (2010), which applied both pre- and post-treatment of cells with resveratrol [44]. However, none of the in vitro studies provided a clear rationale for choosing pretreatment over post-treatment. This seems to suggest that within in vitro models, the focus remains on assessing the overall protective effects of resveratrol and pterostilbene against UV-induced photoaging rather than investigating whether the timing of treatment administration significantly alters the outcome. Without direct comparisons or explicit justifications, it remains unclear whether pre- or post-treatment confers distinct advantages at the cellular level.

### 4.3 Stilbenes and in vivo studies

While in vitro studies give us insight into cellular mechanisms, in vivo applications reveal the true potential of stilbenes. Most studies used resveratrol in animal models to investigate its effects on skin photoaging [[25], [26], [27], [28], [29],^44^,^45^], except for one study that used pterostilbene [29]. There were four routes of administration for resveratrol, whereby 2 studies used topical application [25,29], 3 studies used oral administration [[26], [27], [28]], one study used intradermal injection [44], and one study used subcutaneous injection [45].

For topical application, resveratrol was often encapsulated or formulated to enhance its effects. Arora et al. (2020) applied a topical resveratrol-loaded transethosomal hydrogel formulation to mice, which improved skin grade, increased antioxidants and lowered malondialdehyde (MDA) level. Notably, a conventional resveratrol hydrogel in the same study yielded less impressive results, suggesting that proper formulation is the key to maximising the absorption of resveratrol into the skin [25].

In addition to topical application, three studies also explored the oral administration of resveratrol. While systemic delivery may lack the localised precision of topical treatments, the reviewed studies still demonstrated that orally administered resveratrol can effectively ameliorate skin photoaging. Mostafa et al. (2021) reported improved wrinkling, epidermal atrophy and skin elasticity, alongside increased glutathione (GSH) and selectively modulated autophagy following 50 mg/kg resveratrol daily oral gavage in mice [26]. Another study by Cui et al. (2022) demonstrated that oral gavage of 2 mg/kg resveratrol, administered 3 times a week before irradiation, improved skin structure and hydration, boosted antioxidant activity, and exerted anti-MMP, anti-apoptotic, anti-inflammatory effects while suppressing the mitogen-activated protein kinase (MAPK) pathway [27]. Kim et al. (2019) also found that oral gavage of resveratrol (2, 10, and 50 mg/kg), administered 3 times a week an hour before UVB exposure, ameliorated UVB-induced wrinkle formation, reduced epidermal thickness, showed a dose-dependent increase in antioxidants with a dose-dependent reduction in MMPs [28]. Collectively, these findings emphasise the potential of orally administered resveratrol in combating photoaging from within.

Next, Lee et al. (2010) explored a unique method of resveratrol administration by injecting it intradermally into mice skin 24 hours before UVB exposure. Their approach effectively inhibited MMP-9 and preserved collagen IV levels similar to the control group [44]. Intradermal injections are administered into the dermis and have the slowest absorption time compared to other parenteral routes [55]. It allows for localised and sustained effects directly at the target site. On the other hand, applying this method in real life would require technical skill, making it less practical compared to more straightforward options like topical and oral treatments.

Xia et al. (2024) administered resveratrol to mice via subcutaneous injection two times a week for 8 weeks. The method led to visible improvements in skin appearance both macroscopically and histologically, as well as activated autophagy and AMPK signalling. Hence, it was suggested that AMPK-mediated autophagy aids in repairing skin damage seen in photoaging. Moreover, the administration route of choice was justified, citing that subcutaneous injection was preferred over topical application to ensure better absorption and delivery of resveratrol to the dermis [45]. When compared to humans, subcutaneous injections have the advantage of being self-administered, less painful, and carrying a lower risk of infection. If infection occurs, it tends to remain local rather than systemic. However, delayed response from subcutaneous injections occurs because the drug needs to diffuse from the injection site into the bloodstream [56]. If resveratrol were to be administered subcutaneously, practicality may be an issue as more accessible and non-invasive options like sunscreen are already available in the market to protect the skin.

Sirerol et al. (2015) conducted the only study examining the topical application of pterostilbene in liposomes, comparing its effects to resveratrol in liposomes on mice. Compared to resveratrol, the results showed that pterostilbene more effectively impeded acute UVB-induced increases in skin fold, overall skin thickness, and skin redness. Additionally, mice treated with pterostilbene exhibited no signs of hyperplasia and inflammatory cell infiltration. As for transepidermal water loss (TEWL), both pterostilbene and resveratrol displayed values similar to those of the control group [29]. These findings display the potential of pterostilbene as a topical agent to prevent skin photoaging.

### 4.4 In vivo skin photoaging model considerations

Among the seven in vivo studies reviewed, all studies used mice [[25], [26], [27], [28], [29],^44^,^45^]. Of these studies, only 2 used hairless mice (albino hairless and SKH-1 hairless) [29,44], while the other studies required shaving of the mice (Swiss Albino, CD1, ICR, BALB/c) [[25], [26], [27], [28],^45^]. For shaving methods, all involved shaving the dorsal skin of mice, with an addition of 40 mg/kg ketamine i. m. anesthesia prior to shaving [25], usage of hair removal cream [27] or waxing cream [28]. Although hairless mice eliminate the need for shaving, their higher cost can be a barrier, leading to some studies using mice/rats with fur as a cost-effective alternative, with the additional task of shaving them. When it comes to shaving the mice, it is recommended to anesthetise them to minimise their pain and stress during shaving. If removal creams are used, caution is necessary if the intended treatment is topical. In our review, Arora et al. (2018) [25] shaved mice without additional creams since the treatment was topical, while the studies using hair removal creams administered oral treatments instead.

Next, we explored other criteria for developing a skin photoaging animal model. From the studies reviewed, the age range for the mice/rats was between 4 and 12 weeks old, categorising them as adults. However, one study failed to report the age of the mice used [26], making it difficult to reproduce the results in the future. Regarding the gender of the animal model, 4 studies used female mice [25,26,29,44], and 3 studies used male mice [27,28,45]. Most studies employed the use of a female animal model. This could be due to the less aggressive nature of female mice than their male counterparts. Yet, there have been concerns that the female mice's estrus cycle could affect the outcome of skin photoaging. To definitively ascertain gender as an influencing factor, a study should be done to compare the outcome between male and female mice, which has yet to be carried out to our knowledge. In addition, the link between the estrus cycle and skin photoaging remains unexplored.

The number of animals used per group varied across studies. One study included only 2 animals per group [44], two studies used 6 [25,45], another study involved 8 [28], two studies used 10 animals per group [27,29], and one study utilised the largest sample size with 12 animals per group [26]. Regardless of the number chosen, there was a lack of justification for a specific number of animals selected for each study. Therefore, we recommend that upcoming studies disclose their sample size calculations using either the power analysis [57] or resource equation [58] method to ensure the repeatability and reproducibility of the results.

One key aspect is the UV radiation type used to establish the skin photoaging animal model. Our review highlights interesting trends: 1 study used the full solar spectrum of UV (UVA and UVB) [25], 2 studies used UVA only [26,45], and 4 studies used UVB only [[27], [28], [29],^44^]. UVA that has a longer wavelength, penetrates into the dermis but is less absorbed by DNA, instead exciting other molecules and causing DNA damage indirectly via ROS formation. Despite only reaching the epidermis, UVB is considered the most harmful due to its higher cytotoxicity and genotoxicity, directly causing DNA damage through ROS, ultimately leading to photocarcinogenesis [22,48,[59], [60], [61], [62]]. Consequently, most studies chose UVB to accelerate skin photoaging in animal models due to its more prominent effects on the skin. However, exposing UVA and UVB simultaneously [25] would be more reflective of real-world conditions.

Another key aspect in establishing a skin photoaging model is the duration of UV exposure. The studies reviewed show a wide range. Two studies used a single high-dose exposure of UV, such as Lee et al. (2010) using 180 mJ/cm^2^ of UVB [44] and Sirerol et al. (2015) using 360 mJ/cm^2^ of UVB [29]. Although photoaging is typically caused by repeated and prolonged UV exposure, these studies were included because they drew conclusions linking to photoaging. On the contrary, 5 studies used repeated UV exposure over weeks or months. The shortest duration was 4 weeks. Arora et al. (2018) did not disclose the exact UV doses but used a UV lamp simulating the full solar spectrum, exposing mice once daily for 5 min, 5 times per week for 4 weeks [25]. Whereas Kim et al. (2019) and Cui et al. (2022) followed a 4-week UVB exposure protocol, irradiating mice 3 times a week which brings to a total dose of 1080 mJ/cm^2^ [27,28]. On the other hand, Xia et al. (2024) used UVA starting from minimal erythemal dose (MED) of 0.35 J/cm^2^ (350 mJ/cm^2^), and the doses were increased by 5 % from the day before, for 4 consecutive days of exposure with a 1-day break, in 8 weeks. However, the cumulative dose exposed to the mice was not disclosed in the study [45]. Mostafa et al. (2021) also used UVA over a much longer period (10 weeks) with gradually increasing doses, peaking at 4 MED by Week 4, reaching a total of 8880 mJ/cm^2^ [26].

Three studies [26,29,45] referenced MED, which is defined as the lowest dose of UV that produces visible erythema on the skin after 24 h. While a systematic approach, Gyöngyösi et al. (2016) suggested using a 'clinically relevant dose' (CRD) to account for broader skin reactions like erythema and edema. It was based on findings that C57BL/6 and BALB/c mice were more prone to erythema, while SKH-1 hairless mice predominantly reacted with edema [63]. However, CRD remains underused in current research. Given the variation in methods, animal strains, and UV doses, establishing the MED or CRD for each animal model before experiments could help create a more standardised approach for future studies. Additionally, Xia et al. (2024) argued that frequent high doses of UV irradiation led to acute photodamage in mice, and it is neither reflective nor inconsistent with UV exposure in humans. Hence, the authors proposed a method where researchers should observe the mice's skin for erythema or inflammation 24 h after the previous irradiation. If erythema or inflammation were present, the mice were rested for 2 days. Their approach avoids acute photodamage and is deemed more appropriate for preclinical research on photoaging [45].

When considering the timing of treatment administration, three studies [27,28,44] pretreated animals before UV irradiation, while two studies [25,45] applied treatment post-irradiation. One study [26] did not specify the timing of treatment administration, and only one study [29] directly compared pre- and post-treatment approaches. Sirerol et al. (2015) found that pretreatment resulted in significantly greater protective effects than post-treatment, as evidenced by a significant decrease in skin fold and epidermal thickness in mice. Consequently, the same study proceeded with pretreatment in subsequent experiments [29]. While this is the only study that explicitly compared both approaches, its findings suggest that timing may influence the effectiveness of stilbenes in mitigating photoaging. This highlights the need for further research to determine whether pretreatment consistently yields better outcomes over post-treatment.

### 4.5 Macroscopic and histologic features of photoaging – in vivo

There is a clear consensus on the macroscopic features of photoaging in animal models. Macroscopically, photoaging is characterised by prominent and deep wrinkles [[25], [26], [27], [28], [29],^45^], decreased skin elasticity [26], roughness [27], increased skin hypertrophy [27,45], increased skin fold and overall skin thickness [29], erythema [27,29,45], hyperpigmentation [45] as well as skin peeling and ulceration [27].

However, there are some nuances when it comes to histological characteristics of photoaging in animal models. Collagen and elastin degradation have been described in various ways, including the destruction of fibroblastic collagen bundles, solar elastosis, and disrupted collagen meshwork [25]. Other studies emphasized the fragmentation of elastic and collagen fibres [26], a scattered and fractured arrangement accompanied by a significant reduction in dermal collagen fibres [27], as well as collagen fibres that are degraded, damaged, decreased, disorganised and unevenly distributed within the dermis [45]. Next, across studies, all unanimously agreed on the infiltration of inflammatory cells into the dermis [[26], [27], [28],^45^]. Only one study reported the presence of telangiectatic blood vessels [26]. Notably, only the epidermis showed 2 different outcomes, either epidermal thickening [25,[27], [28], [29],^45^] or epidermal thinning [26]. Both epidermal thickening and thinning can be considered pathological as they represent deviations from normal skin homeostasis. The transition from thickening to thinning of the epidermis is as follows: when keratinocytes in the skin frequently undergo apoptosis due to UV exposure, this causes the formation of ‘sunburn cells’. Epidermal thickening happens when the apoptosis triggers a compensatory wave of increased cell division by surrounding skin cells, to replace the sunburn cells. This response is presumed to protect the basal layer stem cells from further UV damage. Over time, as more UV damage accumulates, the stem cells are eventually lost and result in epidermal thinning (atrophy) [64]. Collectively, the histological characteristics of photoaging can be summarised as follows: collagen and elastin degradation, inflammatory cell infiltration to the dermis, telangiectasia, and structural changes in the epidermis.

### 4.6 Effects of resveratrol and pterostilbene on macroscopic and histologic features of photoaging – in vivo

The effects of resveratrol and pterostilbene on skin photoaging can be examined both macroscopically and histologically. Macroscopically, topical resveratrol loaded in transethosomes improved skin grade, maintained skin elasticity, preserved epidermal integrity with reduced hair follicles, and exhibited negligible inflammation [25]. Oral resveratrol mirrored these benefits by reducing wrinkles [26,28], improving skin elasticity [26], and maintaining a skin surface similar to the negative control group [27]. Meanwhile, resveratrol injected subcutaneously showed reduced erythema and wrinkling [45]. Histologically, collagen improved [27,45], dermal and inflammatory cell infiltration decreased [26,27,45], telangiectasia diminished [26] as well as decreased epidermal thickening [27,28,45] or ameliorated epidermal thinning [26]. The epidermis exhibits one of two outcomes, opposite to those observed in the UV-induced group. If the UV-induced group shows epidermal thickening, the preferrable outcome would be a reduction in thickening. Conversely, if the UV-induced group shows epidermal thinning, the preferable outcome would be the preservation of normal epidermal thickness.

When comparing topical resveratrol to pterostilbene, pterostilbene had the upper hand by preventing increased skin fold, overall skin thickness, and redness more effectively than resveratrol. There was also no hyperplasia and inflammatory cell infiltration in mice treated with pterostilbene [29]. Ultimately, resveratrol exhibits strong photoprotective effects against skin photoaging, macroscopically and histologically. However, further investigation is warranted, as pterostilbene shows better photoprotective effects than resveratrol.

### 4.7 Molecular pathways of resveratrol and pterostilbene towards skin photoaging

**Fig. 2:**
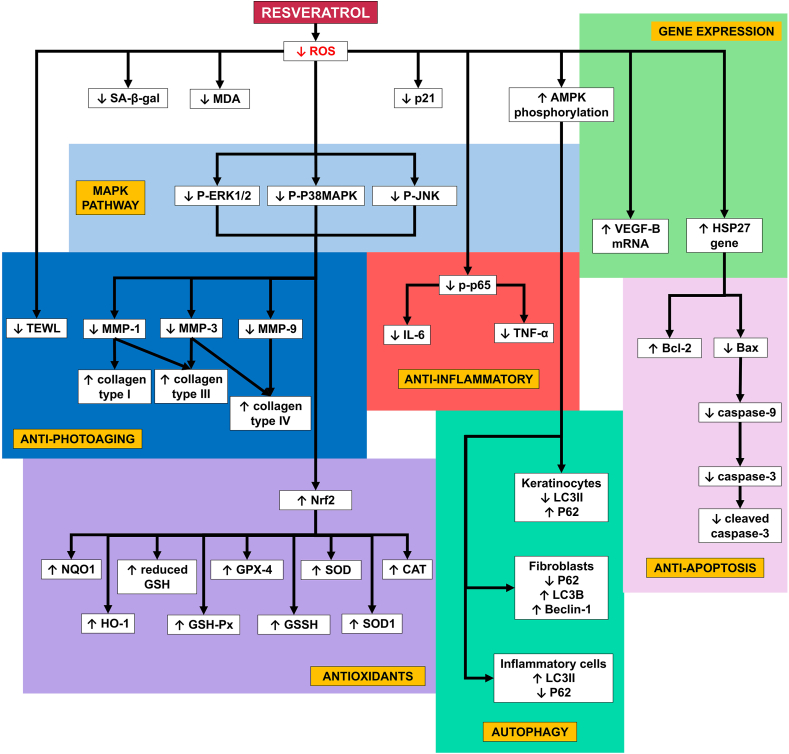
Molecular pathway of resveratrol towards skin photoaging.

**Fig. 3:**
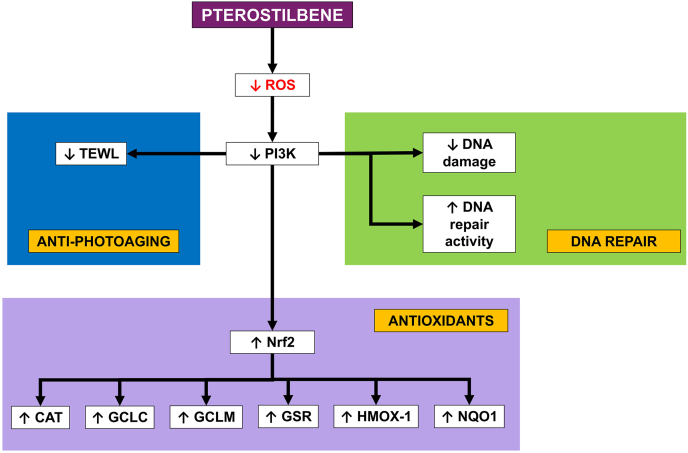
Molecular pathway of pterostilbene towards skin photoaging.

Fig. 2, Fig. 3 elucidate the molecular pathways of resveratrol and pterostilbene towards skin photoaging based on the studies reviewed. Notably, the research on resveratrol is more extensive compared to pterostilbene. To unravel the full potential of both stilbenes, understanding what has been studied thus far regarding skin photoaging is crucial.

### 4.8 Breakthroughs, research gaps and future studies

To date, substantial progress has been made in unravelling the mechanisms behind resveratrol's effects on skin photoaging. Resveratrol holds significant promise as an all-rounder by providing strong protection against skin photoaging both in vitro and in vivo via modulation of anti-inflammatory, autophagy, and apoptosis pathways. Meanwhile, pterostilbene is emerging as an even more promising anti-photoaging compound, potentially surpassing resveratrol. An in vivo study by Kapetanovic et al. (2011) [32] demonstrated that pterostilbene exhibits significantly higher bioavailability (∼80 %) compared to resveratrol (∼20 %) when administered orally. Moreover, total plasma levels of both the parent compound and its metabolites were higher with pterostilbene than with resveratrol. These differences suggest that pterostilbene may exhibit greater biological activity in vivo. Next, the key structural distinction between pterostilbene and resveratrol lies in their functional groups. Pterostilbene possesses two methoxy groups and one hydroxyl group, whereas resveratrol contains three hydroxyl groups. The presence of these methoxy groups enhances the lipophilicity of pterostilbene, leading to improved oral absorption and a greater potential for cellular uptake [31].

Despite these advantages, research on pterostilbene in the context of skin photoaging remains limited. From this review, the only common mechanism that both resveratrol and pterostilbene have been investigated on are antioxidants. However, without direct comparative studies evaluating both stilbenes under the same experimental conditions and dosages, it is difficult to ascertain whether one stilbene is better than the other when it comes to enhancing the antioxidant response in mitigating skin photoaging. Therefore, current findings remain insufficient to establish resveratrol and pterostilbene as definitive preventions or treatments for skin photoaging. Several limitations hinder this process. Firstly, resveratrol is sensitive to light and air which leads to its rapid degradation that complicates its formulation into stable and effective topical treatments [30]. Additionally, current studies lack standardised guidelines regarding UV irradiation duration and cumulative UV dosage needed for skin photoaging to occur in animal models. This inconsistency makes it difficult to determine whether observed anti-photoaging effects are reproducible across different models. Furthermore, none of the selected skin photoaging studies in this review included a positive control, preventing a clear benchmark for evaluating the true efficacy of resveratrol and pterostilbene. To date, topical retinoids have been recognised as the gold standard for photoaging prevention and treatment [65]. In contrast, oral photoprotection lacks an established gold standard, making it difficult to determine its true efficacy [66].

Based on the mapped molecular pathways of both stilbenes, we propose the following targets and mechanisms for future studies. Resveratrol has been widely implicated as a Sirtuin 1 (SIRT1) activator [67,68] which influences multiple cellular processes. However, its exact effects on the sirtuin pathway remain unclear. Sirtuins, or 'longevity proteins', a family of 7 proteins (SIRT1 – SIRT7), are involved in skin pathology, including aging, UV-induced photoaging, and cancer [69]. Despite emerging evidence linking sirtuins to photoaging [48,59,[70], [71], [72], [73], [74], [75], [76], [77], [78]], the connection between sirtuins and stilbenes, such as resveratrol and pterostilbene, remains underexplored.

Another emerging area of interest is the role of miRNAs in photoaging. miRNAs are involved in various biological processes such as epidermal development, proliferation, differentiation, inflammatory responses, immune regulation and wound healing [79]. While only one study has examined the influence of an adipose-derived stem cell extracellular vesicles loaded with a combination of resveratrol, nicotinamide riboside and nicotinamide on miRNA expression [80], other studies suggest a strong link between miRNAs and photoaging [79,81,82]. Thus far, miR-34a, miR145 and miR-383 were implicated in photoaging [82]. Whether both stilbenes modulate specific miRNAs remains uncertain, but deeper insights into their protective mechanisms against skin photoaging would further close this knowledge gap.

In addition, the effects of resveratrol and pterostilbene on skin barrier function remain largely unexplored. Investigating their impact on key skin barrier function markers such as filaggrin, involucrin, and loricrin could provide valuable insights into their potential role in preventing skin dryness and transepidermal water loss (TEWL) associated with photoaging [83,84]. Addressing these gaps in future research will enhance our understanding of the therapeutic potential of resveratrol and pterostilbene in mitigating skin photoaging.

Future studies should prioritise clinical trials and safety evaluations for resveratrol as a topical anti-photoaging agent while extending pterostilbene research into in vivo studies similar to those on resveratrol to enable direct comparisons. Furthermore, there is untapped potential for both resveratrol and pterostilbene to be added to sunscreens to enhance protection against skin photoaging via their antioxidant properties.

Finally, exploring the mechanisms of these stilbenes, both individually and in combination, through in vitro and in vivo studies, as well as human trials, remains essential. All studies included in this review are preclinical, and to date, no clinical trials have been conducted for either stilbene in the context of photoaging. A major challenge in resveratrol clinical trials for other diseases is the poor bioavailability of resveratrol [85]. Hence, efforts should be made to develop resveratrol in different formulations such as nanoformulations, to improve bioavailability and thus applied to photoaging studies. As for pterostilbene, a clinical trial with hypercholesterolemia subjects showed that pterostilbene is generally safe for use in humans at doses up to 250 mg per day [86]. However, the dose from this study does not guarantee efficacy for photoaging and the optimal dose for skin benefits are still unclear. In addition, there is a lack of preclinical studies for pterostilbene to further incentivise the need for clinical trials.

Overall, with the breadth of studies that have been carried out using resveratrol, the next direction would be to move into phase I clinical trials. Unlike pterostilbene with limited in vitro and in vivo studies, more preclinical studies are needed before moving on to clinical trials. This comes alongside exploring alternative modes of administration for both resveratrol and pterostilbene, such as topical or oral routes, to maximise their potential against photoaging. Moreover, there needs to be an active effort to incorporate positive controls in the study design. While topical photoprotection has retinoids as a gold standard, oral photoprotection is still lacking. Therefore, collaborative efforts are needed to establish an oral photoprotection gold standard [66], to evaluate the effectiveness of pterostilbene and resveratrol.

## 5 Conclusion

Current findings strongly suggest that resveratrol shows significant potential in combating skin photoaging. However, establishing a reliable photoaging model with the incorporation of positive controls in the study design is essential before progressing to in vitro or in vivo studies, because no standardised guidelines currently exist. Once a robust foundation of in vitro and in vivo research is built, advancing to human trials becomes critical to validate the efficacy of stilbenes such as resveratrol and pterostilbene. Only through the translational approach can we unlock the full potential of these stilbenes in preventing and treating photoaging.

## CRediT authorship contribution statement

**Raveena Vaidheswary Muralitharan:** Writing – original draft, Visualization, Methodology, Investigation, Data curation, Conceptualization. **Siti Fathiah Masre:** Writing – review & editing, Validation, Supervision, Methodology. **Dayang Fredalina Basri:** Writing – review & editing, Validation, Supervision, Methodology. **Ahmad Rohi Ghazali:** Writing – review & editing, Validation, Supervision, Methodology, Investigation, Conceptualization.

## Funding

The study was funded by Universiti Kebangsaan Malaysia through the Research University Grant (GUP-2020-053).

## Declaration of competing interest

The authors declare that they have no known competing financial interests or personal relationships that could have appeared to influence the work reported in this paper.

## Data Availability

Data will be made available on request.
